# The demographic decline of a sea lion population followed multi-decadal sea surface warming

**DOI:** 10.1038/s41598-020-67534-0

**Published:** 2020-06-26

**Authors:** Karen Adame, Fernando R. Elorriaga-Verplancken, Emilio Beier, Karina Acevedo-Whitehouse, Mario A. Pardo

**Affiliations:** 1 Centro de Investigación Científica y de Educación Superior de Ensenada (CICESE), Unidad La Paz, Laboratorio de Macroecología Marina, 23050 La Paz, Baja California Sur Mexico; 2 Instituto Politécnico Nacional, Centro Interdisciplinario de Ciencias Marinas, 23096 La Paz, Baja California Sur Mexico; 3 Autonomous University of Queretaro, School of Natural Sciences, Unit for Basic and Applied Microbiology, 76230 Queretaro, Mexico; 4 Consejo Nacional de Ciencia y Tecnología (CONACYT) – CICESE, Unidad La Paz, Laboratorio de Macroecología Marina, 23050 La Paz, Baja California Sur Mexico

**Keywords:** Climate-change ecology, Climate-change ecology, Marine biology, Marine mammals, Ecological modelling, Macroecology, Population dynamics

## Abstract

The population growth of top predators depends largely on environmental conditions suitable for aggregating sufficient and high-quality prey. We reconstructed numerically the size of a resident population of California sea lions in the Gulf of California during 1978–2019 and its relation with multi-decadal sea surface temperature anomalies. This is the first multi-decadal examination of the sea surface temperature of the Gulf of California and of one of its major predators. A three-decade sustained warming explained the population’s trend accounting for 92% of the variance, including a 65% decline between 1991 and 2019. Long-term warming conditions started in the late 80s, followed by the population’s decline from 43,834 animals (range 34,080–58,274) in 1991 to only 15,291 (range 11,861–20,316) in 2019. The models suggested a century-scale optimum sea surface habitat occurring in mildly temperate waters, from 0.18 to 0.39 °C above the 100-year mean. The mechanistic links of this relation are still untested, but apparent diversification of pelagic fish catches suggests a reduction of high quality prey. We propose this population should be considered vulnerable to any disturbance that could add to the negative effects of the current warm sea surface conditions in the Gulf of California.

## Introduction

The physical structure of oceanic habitats often determines, through a multi-step process, the success of animal populations^1^. Sea surface temperature and its variability is widely known to affect oceanic top predators such as pinnipeds, through bottom-up mechanisms^2–4^. Abrupt or sustained changes of sea surface temperature affect the abundance and diversity of plankton communities^5^, pelagic fishes^6^, and ultimately marine mammals^7,8^. The latter typically respond with alterations in foraging habits^9,10^, key physiological processes^11,12^, reproductive success, or survival^4^. Although many marine mammal populations can withstand and recover from short-term sea surface warming conditions (e.g. El Niño)^13^, sustained positive environmental trends at a multi-decadal scale typically cause important shifts in the base of marine ecosystems^14^, and arguably the diversity of potential prey. These shifts could lead to the decline of some marine mammal populations as the conditions move away from the optimum habitat to which the species have adapted^15^.

In the Gulf of California (hereafter “the gulf”), there is a resident population of California sea lions (*Zalophus californianus*)^16^ genetically isolated from the other populations of the Northeast Pacific Ocean^17^. Although a ~ 20% reduction of this population during the 1990s has been proposed based on partial counts spanning 1997–2004^18,19^, its total size and temporal trend, as well as the historical environmental context, remain unknown. A recent review mentioned that the population decreased 44% between 1979 and 2016, based on unpublished data^20^. Negative interaction with fisheries, and a temporal decline of the Pacific sardine (*Sardinops sagax*) in the early 1990s, as a result of unspecified environmental changes, have been proposed as potential causes of the apparent decline^19^. Nevertheless, interannual events such as El Niño do not have a consistent correlation with the dynamics or health indicators of the gulf’s population^21,22^. Given the uncertainty in both the apparent population decline and its drivers, we hypothesized that a long-term sea surface warming in the Gulf of California could be related, since this environmental variable has the potential to trigger progressive changes in the base of marine ecosystems, arguably altering the composition and diversity of prey for top predators.

**Figure 1:**
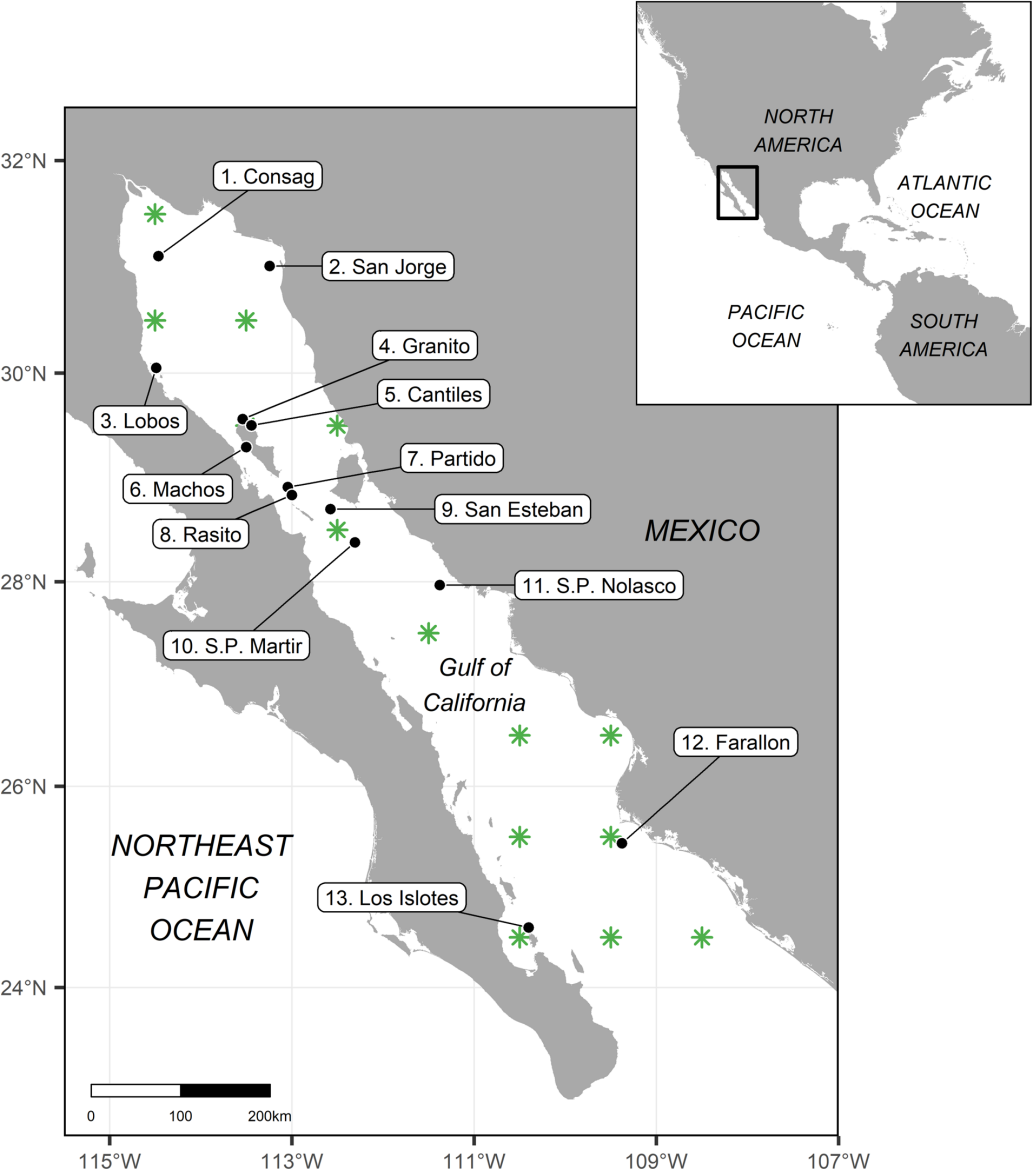
The Gulf of California in the Northeast Pacific Ocean. Black dots are the locations of the 13 reproductive colonies of California sea lions (*Zalophus californianus*), numbered from north to south. The green asterisks are the locations of the predicted sea surface temperature data used in this study. The map was created with the package “ggplot2” (https://cran.r-project.org/web/packages/ggplot2/index.html) in R (https://www.r-project.org/).

To find support for this hypothesis, we made a numerical reconstruction of the California sea lion total population size and its changes during the last 42 years (1978–2019), using individual trends of the 13 reproductive colonies within the Gulf of California (Fig. 1), inferred from all available animal counts. Then, we explored the multi-decadal variability of the gulf’s sea surface temperature during the last 100 years (1920–2019), from which we were able to predict successfully the population’s multi-decadal dynamics. Nevertheless, it is important to point out that the lack of information did not allow us to test for the mechanisms involved in such habitat-species relationship, and therefore we can only propose hypothetical ecological scenarios to explain it based on similar responses already described for interannual or multiannual warm conditions.

The reproductive colonies in the Gulf of California are, from north to south: Rocas Consag, San Jorge, Lobos, Granito, Los Cantiles, Los Machos, El Partido, El Rasito, San Esteban, San Pedro Martir, San Pedro Nolasco, Farallon de San Ignacio, and Los Islotes^16,23^. Even though they are often grouped into four ecological regions^23,24^, or three genetic groups^17^, they function as individual units for the most part, because California sea lion individuals commonly exhibit natal philopatry to their colonies^22^, which also reflects distinctive foraging habits^25^. Our results confirmed a dramatic population decline in the last 28 years (1991–2019), after reaching its maximum in the early 1990s. The gulf’s annual sea surface temperature anomalies from the 100-year mean (1920–2019) were able to predict the population size dynamics at a multi-decadal level and suggested an optimum habitat at mildly temperate conditions.

## Results

**Figure 2:**
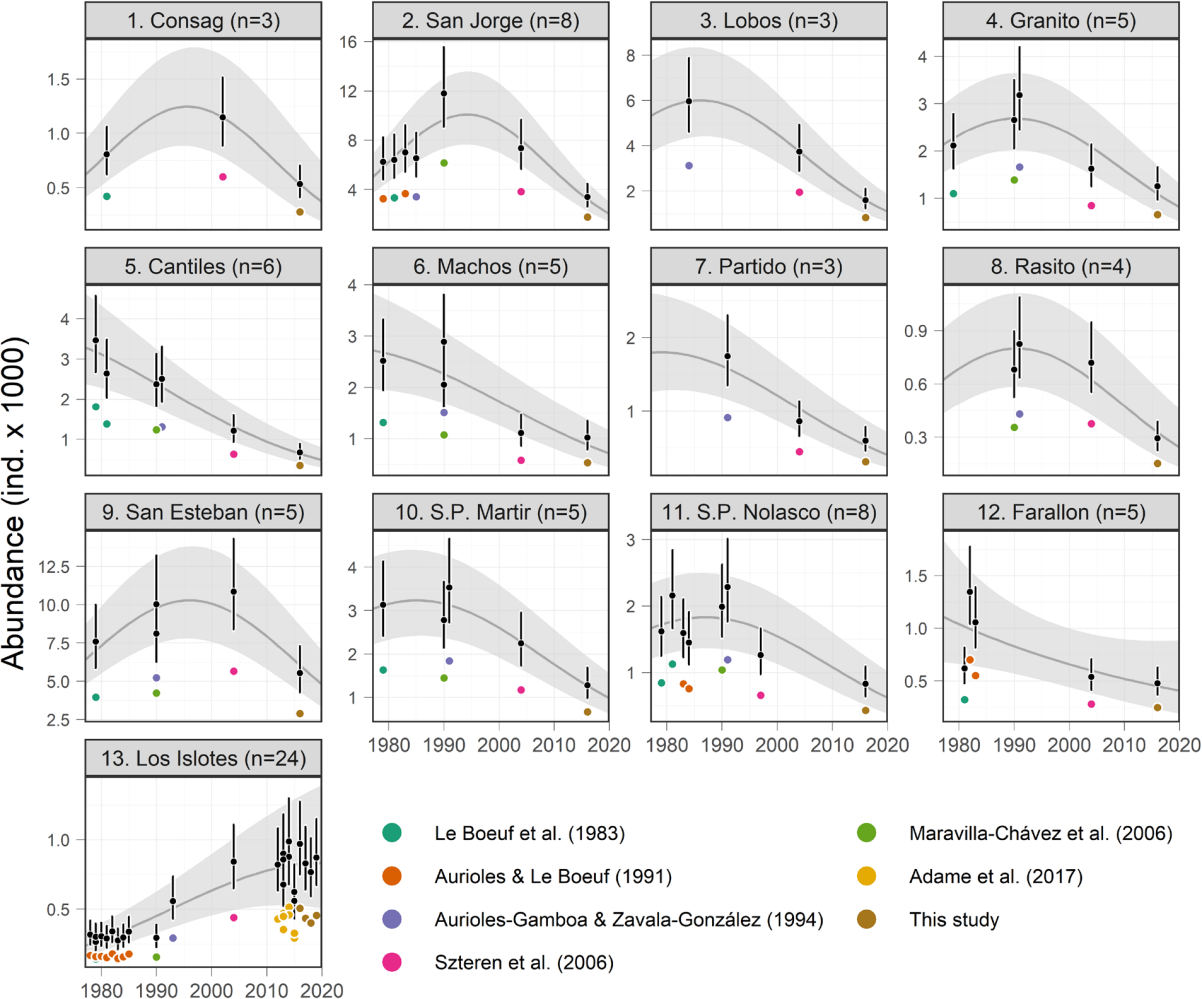
Abundance trends of California sea lions (*Zalophus californianus*) in the Gulf of California at the 13 reproductive colonies. Colored dots represent the original boat-based counts with the reference that reported them. Black dots and error bars represent the medians and the 95%-credible intervals (CIs) of the estimated abundance, accounting for perception and availability biases. Medians and 95%-CIs of the predicted trend are shown as dark gray lines and shaded areas, respectively. The colonies are numbered from north to south as portrayed in Fig. 1, along with their number of abundance estimates (n).

For all the 13 reproductive colonies, a second-order polynomial regression was the best model describing the abundance as a function of the year at a multi-decadal scale, over the simple linear trend. The mean drone-based perception bias correction factor added 41% (range 33–48) to the original boat-based counts. The mean percentage of non-pups in the population was 80% (range 77–82), to which a 40.5% (range 4.3–93.2) was added to account for animals likely at sea (Table 1). Almost all reproductive colonies exhibited a decreasing trend during the last three decades (1990s–2010s), except for the southernmost small colony Los Islotes, which showed a sustained population growth in that period, with an apparent stabilization in the last 4 years (2015–2019) (Fig. 2). Some colonies showed decline even since 1978, but most of them, including the largest ones, showed a bell-shaped trend, with an increase during the 1980s followed by a sustained decrease since the early 1990s.

**Table 1:** Statistical summary of the posterior distributions of the most relevant parameters estimated by the models.

Parameter	In JAGS code	Mean	SD	2.5%	25%	50%	75%	97.5%	$\hat{R}$	*N*_*eff*_ (%)
**Numerical reconstruction of the population size**										
Population size in 1978 (1st of the series)	pred_sum_gulf^1^	37,651	5,271	28,728	33,811	37,139	41,112	48,755	1.001	100
Population size in 1991 (highest peak)	pred_sum_gulf^14^	45,970	6,428	35,068	41,281	45,333	50,192	59,518	1.001	100
Population size in 2019	pred_sum_gulf^42^	16,006	2,245	12,217	14,371	15,787	17,480	20,736	1.001	100
1980s increase (%)	diff_decade_perc^1^	15.570	0.918	13.784	14.947	15.571	16.190	17.373	1.001	83
1990s decrease (%)	diff_decade_perc^2^	− 10.334	0.288	− 10.896	− 10.528	− 10.335	− 10.140	− 9.769	1.001	100
2000s decrease (%)	diff_decade_perc^3^	− 31.994	0.378	− 32.736	− 32.247	− 31.995	− 31.738	− 31.252	1.001	100
2010s decrease (%)	diff_decade_perc^4^	− 39.711	0.500	− 40.685	− 40.051	− 39.713	− 39.375	− 38.725	1.001	100
28-year decrease (%) (1991–2019)	diff_max_min	− 65.179	0.522	− 66.196	− 65.532	− 65.183	− 64.828	− 64.149	1.001	100
Proportion of undetected animals	prop_non_det	0.444	0.004	0.436	0.441	0.444	0.446	0.451	1.001	35
Proportion of non-pup animals	mu_prop_non_pups	0.798	0.010	0.777	0.791	0.798	0.805	0.818	1.001	73
Proportion of non-pups at sea	mu_at_sea_prop	0.440	0.236	0.039	0.268	0.417	0.595	0.939	1.001	92
Proportion at non-reproductive colonies	prop_other_rook	0.060	0.002	0.057	0.059	0.060	0.061	0.063	1.001	72
**100-year analysis of sea surface temperature (SST)**										
Mean SST	centu_mean	22.241	0.109	22.026	22.167	22.241	22.314	22.455	1.001	100
Mean SSTa	mu_sst_anom	0.497	0.017	0.464	0.485	0.497	0.508	0.530	1.001	100
**Ecological model (population size response to SSTa)**										
Intercept	a0	3.773	0.034	3.705	3.751	3.774	3.796	3.838	1.001	72
First coefficient	a1	35.987	48.552	0.846	3.168	12.379	48.897	174.368	1.006	0.38
Second coefficient	a2	− 1.367	0.178	− 1.714	− 1.487	− 1.368	− 1.247	− 1.016	1.001	17
Mixing parameter	theta	0.824	0.236	0.137	0.756	0.938	0.984	0.996	1.004	0.72
Bayesian R-squared	r_squ_ecol	0.915	0.012	0.881	0.910	0.918	0.923	0.927	1.001	100

The names in JAGS language are those of the code in “Methods” section. A Gelman–Rubin statistic ($\hat{R}$) close to 1 indicates good convergence of chains. A high percentage of the effective number of iterations (*N*_*eff*_) indicates less uncertainty in the parameter’s estimation.

**Figure 3:**
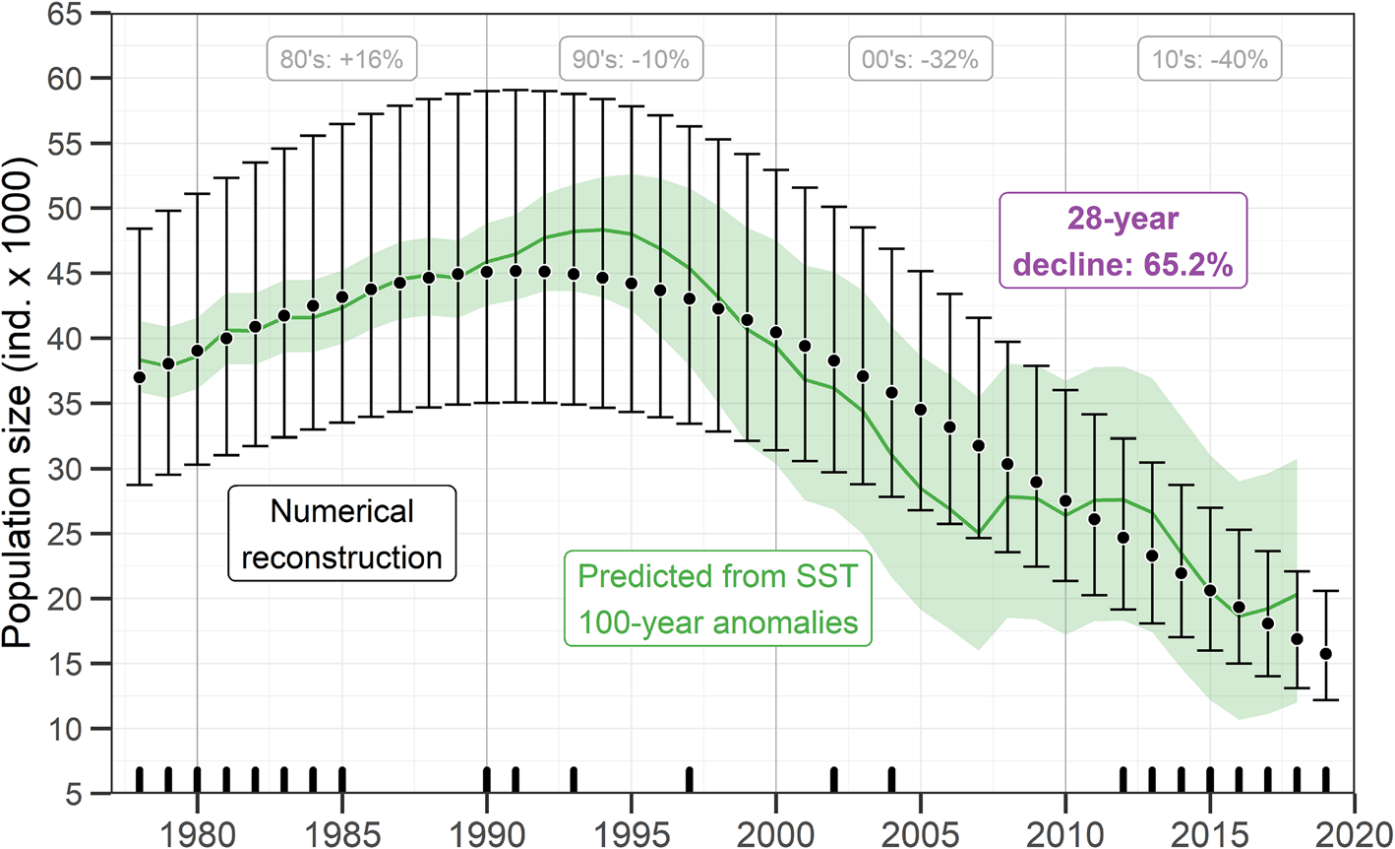
Historical population size of California sea lions (*Zalophus californianus*) in the Gulf of California. Annual medians (black dots) and 95%-credible intervals (CI; black error bars) of the numerical reconstruction are shown. Black inner ticks on the X axis mark the years for which any colony count was available. Medians of decadal changes and the 28-year decline in percentage are within gray and purple boxes, respectively. The population size prediction from the multi-decadal anomalies of sea surface temperature (SST) is shown as a green thick line (median) and green shaded area (95%-CI).

The most populated colonies predicted for 2019 were San Esteban with 4,959 animals (range 3,308–7,633), San Jorge with 2,236 animals (range 1,476–3,476), and Lobos with 1,175 (range 677–2,100). The smallest were El Rasito, Partido, and Consag, with abundances of less than 500 animals. The sum of the colonies’ annual predictions was augmented by 6% (range 5.7–6.3), corresponding to the estimated proportion of animals at the 16 non-reproductive colonies during a typical breeding season (Table 1). According to this numerical reconstruction, the population had 35,972 animals (range 27,892–47,682) in 1978. During the 1980s, it gained 15.5% (range 13.7–17.4), reaching its historical maximum in 1991 with 43,834 animals (range 34,080–58,274). From that peak, the population lost 10.3% (range 9.8–10.9) during the 1990s, 32% (range 31.3–32.7) in the 2000s, and a 39.7% (range 38.7–40.7) in the 2010s. For 2019, the population size would have been of 15,291 animals (range 11,861–20,316), which represents a 65.2% decline (range 64.1–66.2) in the last 28 years (1991–2019) (Fig. 3; Table 1).

**Figure 4:**
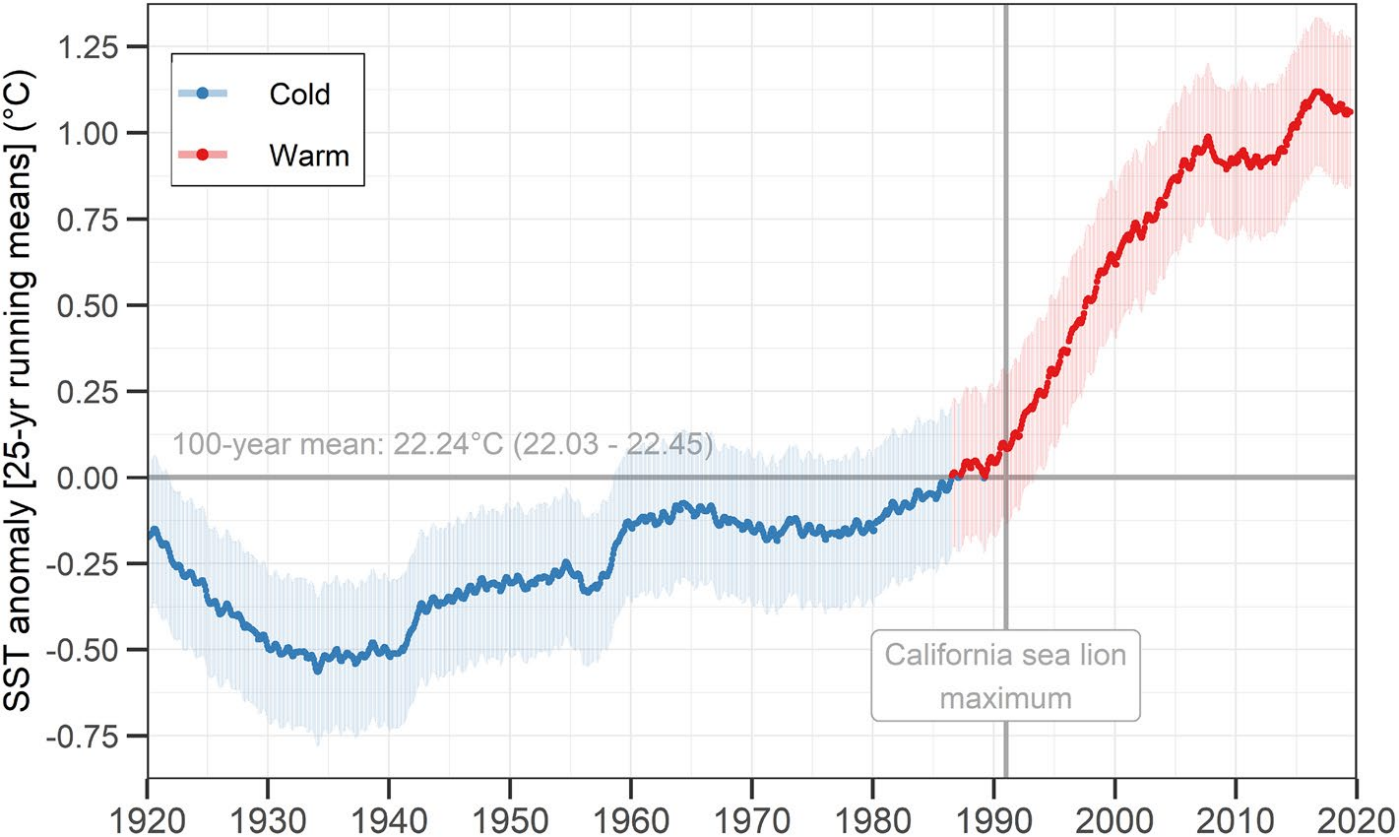
Sea surface temperature (SST) anomalies from the 100-year mean (horizontal gray line) in the Gulf of California. The colored thick line represents the median and the shaded area is the 95%-credible interval. The locations of SST estimations are portrayed in Fig. 1. The maximum California sea lion (*Zalophus californianus*) population size occurred in 1991 (Fig. 3).

The time series of sea surface temperature anomalies (SSTa) from the 100-year mean of 22.2 °C (range 22.02–22.45 °C) showed a warming of the Gulf of California since the late 1980s (Fig. 4). It was especially steep during the 1990s, and less accelerated during the 2000s and the 2010s, reaching + 1.06 °C in 2019 (range + 0.85 to – 1.28). The minimum temperatures occurred during the mid-1930s, reaching anomalies of − 0.57 °C (range − 0.77 to − 0.35). During 1991, when the California sea lion population size reached its maximum, the median SSTa was + 0.11 °C (range − 0.11 to + 0.31) (Fig. 4).

**Figure 5:**
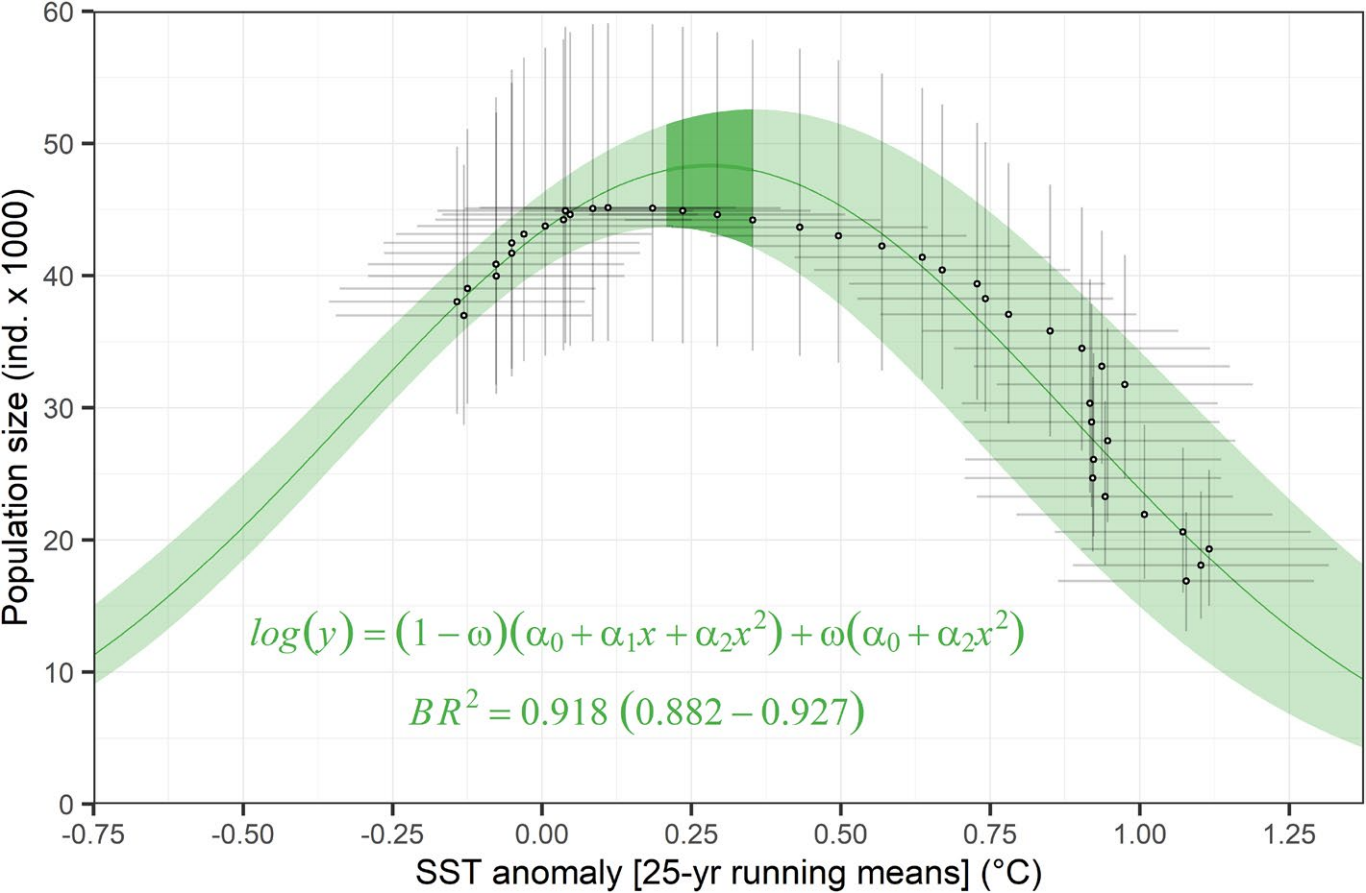
The California sea lion (*Zalophus californianus*) population size in the Gulf of California as a function of sea surface temperature (SST) multi-decadal anomalies from the 100-year mean of 22.24 °C (range 22.02–22.45). The green line and shaded area are the median prediction and the 95%-credible interval (CI), respectively. The darker line and shaded area represent the multi-decadal habitat optimum, between + 0.17 and + 0.40 °C. Black dots and thin error bars are the medians and 95%-CIs of the annual estimations of both variables. The posterior summaries of the equation’s parameters are portrayed in Table 1.

According to our model, the population size of California sea lions in the Gulf of California can be predicted successfully from the SSTa at a multi-decadal scale (i.e. 25-year running means). After testing for several degrees of curve complexity, the best model describing this relationship was a mixture between a second-order polynomial and a parabola function. This was accomplished through a mixing parameter, whose median closer to 1 (0.77; range 0.06–0.99) indicated that the parabola function contributed with most of the fit (Table 1). The proportion of the variance explained by this model (i.e. the Bayesian R-squared) was 0.918 (range 0.880–0.926), suggesting an extremely high predictability. The shape of the resulting curve (Fig. 5) implies that the population size is expected to decrease at both very high and very low SSTa. According to the predictions of this model (median and 95% CI), the range at which the population size would reach its maximum would be during anomalies from + 0.17 to + 0.40 °C (i.e. SST means of between 22.49 and 22.72 °C) (Fig. 5). This could be interpreted as the population’s optimum physical habitat conditions. The model predictions also indicated that above this optimum, the population size would have reached half of its maximum at an anomaly of + 0.76 °C from the 100-year mean (i.e. around 23 °C).

The annual population size estimates using the posterior coefficients of the habitat model (Table 1) confirmed its high predictability. Though with higher uncertainty for the last two decades (i.e. during warmer conditions), the model predicted successfully the median population sizes that resulted from the numerical reconstruction (Fig. 3). Moreover, since this ecological model was based on SST data at a higher temporal resolution than that of the animal counts, it was also capable of showing some details in the expected trend that the numerical reconstruction could not. The most important difference was that the numerical reconstruction tended steadily towards lower values, whereas the abundances predicted from the SSTa suggested some level of stabilization of the trend during the late 2000s and the 2010s, or at least, a less pronounced decrease, compared to that of the 1990s and early 2000s (Fig. 3).

## Discussion

The negative relation between the multi-decadal SST warming in the Gulf of California (1990s–2010s) and the California sea lion population trend was evident. This environmental condition predicted accurately the annual population size and trend, accounting for 91.8% of the variability (range 0.882–0.927). Such high explained variance could respond to the simple opposite trends of the SSTa means and of the reconstructed sea lion population size (based on the sum of the colonies’ second-order polynomials), which represent the multi-decadal scale, and therefore such relation would be much variable at shorter interannual or multiannual scales.

California sea lions reflect long-term changes in the marine environment^4,26^ because, as marine mammals and top predators in general, they are conspicuous, with high energetic demands, and have a long lifespan^3^. Therefore, it is possible that other predators within the gulf have also reacted to this scale of environmental variability. Such effects at the multi-decadal scale cannot be interpreted as equivalent to those that have been observed in response to inter-annual warm events such as El Niño, which are well described^21,27, 28^, and typically include a strong generalized depletion of prey availability and drastic changes in foraging habits^10,29,30^. Compared to these short-term shifts, the knowledge of the effects of long-term environmental changes is very limited^31–33^, yet it is essential to understand and predict population dynamics under climate change scenarios.

While the negative effects of El Niño events tend to last for only 2 or 3 years^21,29^, a multi-decadal warming would suppose a progressive, sustained, habitat change affecting multiple generations. It is challenging to understand the mechanisms involved in the response of a predator population to this scale of sea surface warming, to the point of reducing dramatically its size. One of the possible causes of a long-term decline in a spatially restrained pinniped population could be a diet change from high quality prey to what has been referred to as “junk food”^9,32^. When preferred prey becomes less available, predators must resort to a less nutritious diet. In the California Current Large Marine Ecosystem, California sea lions feed commonly on small pelagic fishes, including several species of sardine (*Sardinops sagax*), anchovy (*Engraulis mordax*), and mackerel, which are considered of high quality because of their calorie contents that range from 1.31 to 2.17 cal g^-1^, and total fat contents from 0.048 to 0.124 g^−1^, in comparison to other prey more common during warming conditions, such as the market squid (*Doryteuthis opalescens*; 0.92 cal g^−1^; 0.014 total fat g^−1^) or the shortbelly rockfish (*Sebastes *sp.; 0.94 cal g^−1^; 0.016 total fat g^−1^)^9,34^. In that region, this type of prey replacement has resulted in pups with lower body masses. Nevertheless, in a higher latitude environment off Alaska, a more diverse diet has been related to a less pronounced population decline for Steller sea lions (*Eumetopias jubatus*)^33^, which suggests that each predator can react differently to prey composition and availability depending on the type of oceanic habitat and the species’ particular requirements.

An analogous prey replacement could have happened in the Gulf of California in 1978–2019, although this California sea lion population has different foraging habits than those of the California Current Large Marine Ecosystem, and few studies have described its diet^25,35^. It varies between colonies and consists on a large variety of species, including high-trophic level items such as the midshipman (*Porichthys* sp.) and squid (*Leachia* sp.), as well as low-trophic level species like the Pacific jack mackerel (*Trachurus symmetricus*), the Pacific sardine (*Sardinops sagax*), and the northern anchovy (*Engraulis mordax*). These prey were found in scats collected in the mid 1990s and early 2000s, so the composition of the diet in the late 1980s, before the population decline, is unknown. However, some trends in the fishery of small pelagic species could support the idea of a shift in the base of the trophic web. Sardine fisheries in the Gulf of California increased slowly until 1989; then, a massive collapse occurred. The catches plummeted from almost 300,000 tons to 7,000 tons in less than 3 years. Since then, several rises and falls have occurred, including the most extreme in 2008, when catches fell from over 500,000 tons to 3,000 tons in 5 years^36^. Nevertheless, as sardine fisheries plummeted and their trend became more unpredictable, other small pelagic fishes such as the thread herring (*Opisthonema libertate*), the Pacific mackerel (*Scomber japonicus*), the northern anchovy, and the bigmouth sardine (*Cetengraulis mysticetus*), gained relative importance in the catches^37^. This could indicate a diversification of the potential prey for California sea lions as the sea surface temperature increased. The effect of ocean surface warming on global fisheries is well documented, particularly in temperate regions, where warmer water species are being captured at higher latitudes^38,39^. Nevertheless, since the coverage of available data of potential prey is not comparable to the multi-decadal scale of this study, nor is spatially representative of the entire gulf, and given the high heterogeneity of fishing effort, it is not possible to validate this hypothetical ecological mechanism.

If California sea lions have been foraging on more diverse but lower quality prey in the Gulf of California for several generations, it is expected that the main drivers of the population dynamics, fertility and/or survival rates, would have lowered. Although reductions in both parameters are common consequences of short-term warm anomalies^9,13,31^, their link to multi-decadal environmental shifts is unknown. Nevertheless, the mean proportion of pups during boat-based counts before the decline (1979–1991) was ~ 25%, and ~ 42% for adult females^18^. In 2016, we found a proportion of pups of only 15%, whereas the proportion of adult females was similar (~ 42%) (Supplementary Information, Table S1), suggesting a reduction in fertility or pup survival that should be further explored. In contrast, in a colony of the species in the California Current Large Marine Ecosystem, with a positive population trend until 2014, the latest proportions of pups and adult females were ~ 41% and ~ 40%, respectively^40^.

The California sea lion population off the U.S. seems to have grown close to its estimated carrying capacity^8^. Although we do not have enough information to estimate directly that parameter for the gulf’s population, it could be close to the maximum population size reached in 1991, according to our model (Fig. 3). This could be addressed more accurately in the future by studying other external sources of pressure regulating fertility and survival besides the environmental conditions suitable for aggregating enough and high-quality prey. The fact that both populations present opposite demographic trends would reflect the differential effects that a multi-decadal ocean warming could pose given particular oceanographic dynamics and the availability of different types of prey. Given the genetic isolation between both populations^17^ and the lack of connectivity between them, we do not expect their opposite trends are related. The exception to this could be the gulf’s southernmost small colony Los Islotes, which showed a positive multi-decadal trend (Fig. 2), apparently reaching its carrying capacity^22^, and for which some connectivity with colonies in the southern California Current Large Marine Ecosystem could occur^41,42^.

An increased foraging effort by juvenile and adult animals in warmer conditions should not be discarded as a complementary factor that could explain the decline of California sea lions on land. During warm anomalies, telemetry studies have shown that this species displays offshore foraging trips up to three times longer, presumably as a consequence of reduced prey availability^43,44^, resulting in fewer animals on land when counts are made. Further telemetry data are needed to build a dynamic correction factor of animals likely at sea. Increasing foraging effort can also result in decreased pup survival^13,30^ by reducing lactation periods. Nevertheless, a progressive decrease in prey quality rather than quantity would not necessarily imply increase in adult females’ foraging duration, but rather a reduction in the quality of their milk throughout several generations.

The 15.5% increase (range 13.7–17.4) of the population size during the 1980s (Fig. 5) was also successfully predicted by the SSTa. Since the shape of the curve of that model was unimodal, the population size would also be lower under extreme cold anomalies at the multi-decadal scale. The optimum sea surface temperatures revealed by this model could hypothetically benefit the occurrence of high quality prey in the Gulf of California, such as sardine, whose fishery also increased during the 1970s and early 1980s^36,45,46^. Though it could be argued that this California sea lion population increased during that period as a result of its recovery from documented legal hunting prior to the 1970s^20, 47,48^, there is no evidence that the level of hunting was high enough to produce an appreciable decline of the population from which it would have recovered, at least not enough for a bottleneck effect^49^. Unfortunately, without more information about the hunting numbers, the possibility that the population growth was a co-occurrence of a recovery process and a progressively more suitable environment for the species is untestable at this point.

One particular finding of this study, and the exception to the general pattern, was the abundance trend at the colony Los Islotes, which was the only one that showed a steady increase during the study period, tending to stabilize in the last 5 years. It is the southernmost reproductive colony of the Gulf of California, located in La Paz Bay, a region characterized for its high biological production year round, attributed to a local mesoscale gyre phenomenon occurring during summer^50,51^, which would provide a more seasonally stable prey availability for the colony.

More studies focused on the foraging habits and reproductive health of the population, as well as studies on the abundance and diversity of potential prey, are needed to reach satisfactory conclusions on the mechanisms driving the population’s long-term dynamics. It is also probable that other predators in the Gulf of California that depend on similar low-trophic-level prey would show similar population trends, but to our knowledge, this is the first study quantifying such a long-term temporal scale of environmental variability and biological response in the gulf. Based on our results, we propose that the California sea lion population of the Gulf of California should be considered as vulnerable to any disturbance that could add to the apparent negative effects of the current sea surface warming conditions. Such conservation status should be maintained until the population size reaches at least half of the maximum observed during the early 1990s, which our models predict would occur at a maximum SST anomaly of + 0.76 °C from the 100-year mean.

## Methods

All the analyses described below were based on algebraically explicit Bayesian regression models^52,53^, whose parameters of interest were estimated as samples from their posterior distributions through a Markov Chain Monte Carlo procedure, implemented in the language Just Another Gibbs Sampler (JAGS)^54^ in R^55^. This approach avoids the loss of information through the propagation of uncertainty between connected estimations and models, accounting effectively for scarce and/or sparse data, if needed. We ran one million iterations in five independent chains, retaining every 20th value to avoid autocorrelation, and discarding the first 20% as a burn-in phase for each chain (see detailed algebraic explanations and JAGS codes below). The results of the estimations are reported throughout the text as their medians, accompanied by ranges that represent the 95%-credible intervals of the posterior distributions. All the data needed to run the models is provided fully arranged as three R’s data list objects in the Supplementary Information File S1.

### Population abundance and trend

When estimating abundance of pinnipeds, pup counts are typically used to infer those of other age classes from life charts that take into account life expectancy and fertility rates, among other parameters^8,56,57^. Unfortunately, only part of the available historical counts of California sea lions in the Gulf of California contains information on pups (Supplementary Information Table S1), and there are no life charts specifically estimated for each reproductive colony^20^; therefore, such traditional estimates were not feasible. Instead, we focused on counts of all age/sex categories (i.e. total counts), as a consistent parameter that can be also used to estimate pinniped abundance^18,19,40^, especially if some perception and availability biases are accounted for^58,59^. For this, we used all counts available in the literature^16,18,19,21,59,60^ and new, reported here for the first time.

The main challenge for estimating the population size of California sea lions and its multi-decadal trend in the Gulf of California was that there is only one breeding season, in 2016, for which there are available counts of animals in all of the 13 reproductive colonies, an effort of this study for which we implemented both visual and drone-based counts. The rest of the available counts spanning the 42-year time series (1978–2019), both published and described here for the first time, were made separately for different colonies during different breeding seasons, spanning 1978–2019. Therefore, it was impossible to estimate more than one total abundance based on the simple sum of counts from all colonies. Fortunately, although very sparse in time, these 86 counts followed the same protocols^16,18,19,59^, and therefore were comparable. This method consists in circumnavigating the colonies during morning hours, at 15–45 m from the shoreline, with hand-held binoculars, at a speed of 5–7 km h^−1^. The counts included all animals detected on land, as well as those swimming near the surface between the boat and the colony.

To solve perception bias and to correct categorization errors, 16 drone-based counts were made parallel to the boat-based counts during the 2016 breeding season (Supplementary Information Table S1), taking aerial photographs of all the areas with presence of California sea lions (see detailed protocol in Adame et al.^59^). The categorization of California sea lions followed established guidelines for identifying each individual as adult male, sub-adult male, adult female, juvenile, pup, or undetermined^16,61,62^. The counts reported for the first time in this study were made during 2016–2019 under research permits SGPA/DGVS/00050/16-19 issued by *Dirección General de Vida Silvestre*—*Secretaría de Medio Ambiente y Recursos Naturales*, with authorization of *Comisión Nacional de Áreas Naturales Protegidas*.

The first step of the analysis was fitting individual regressions of animal boat-based counts at each reproductive colony as functions of the year (1978–2019), only during breeding seasons. We tested for a simple linear trend and a second-order polynomial, choosing the best fit according to the lowest value of the Deviance Information Criterion (DIC)^63^. A Poisson likelihood was stated for all counts with a logarithmic link function (see model equations below). We used all published counts, and those reported for the first time in this study (Supplementary Information, Table S1). Given that most of the counts were very sparse in time during the 42-year study period (1978–2019), these curves were intended to identify multi-decadal variations only, rather than interannual or more fine-scale dynamics. However, the Bayesian framework of the analyses assured that the uncertainty associated with the estimations captured accurately the number of observations and their sparsity in time, which was the especial case of Consag, Lobos, and Partido.

The perception bias of boat-based counts was estimated for all available surveys as the proportion of animals detected from the boat with respect to those detected from the aerial drone photos at each reproductive colony. All proportions were stated as binomial likelihoods, whose inverse main parameters were used as additive correction factors for the annual abundance predictions. We also estimated an availability bias correction factor to add the proportion of animals likely to be at sea when the surveys were made. For that, we used a mean of those proportions reported in a study that compared on-land and at-sea aerial counts at colonies of the same species in the California Current Large Marine Ecosystem^58^. Unfortunately, there was no available information for our study area for this purpose. Since those proportions were with respect to all categories except pups, we had to estimate first the mean proportion of non-pup animals in the Gulf of California from 76 counts with available information (Supplementary Information, Table S1). Then, the correction factor was applied only to non-pup animals for each reproductive colony. Although the proportion of animals foraging at sea can vary as a function of prey availability^43,64^, there was no available data or previous information that allowed us to address this dynamically. Therefore, our model assumed this proportion as constant. Since El Partido and El Rasito did not have enough observations during the first 10 years of the time series, and given that both colonies have very limited area available for California sea lions, we set upper truncation limits for their predictions at the beginning of the time series to their maximum total counts available.

To obtain the annual posterior distributions of the total population size, we summed the annual predictions of abundance at each reproductive colony from 1978 to 2019, all within the same hierarchical Bayesian structure to propagate the uncertainties of the colonies’ estimations^53^. We also added a final availability bias correction factor to account for the animals likely present at the 16 known non-reproductive colonies during a typical breeding season, based on the counts reported by the only study that included such colonies^18^. To better visualize the population size dynamics, we also estimated decadal and maximum percentages of change during the 42-year time series.

### 100-year anomalies of sea surface temperature

We estimated 25-year running means of sea surface temperature within the Gulf of California and their anomalies from the 100-year mean, from 1920 to 2019 (n = 1,193). This allowed us to filter interannual and decadal signals such as El Niño Southern Oscillation (ENSO) or the Pacific Decadal Oscillation (PDO), respectively, keeping only multi-decadal variability in accordance to the scale of the California sea lion population trend explored in this study. The dataset consisted of predictions from a two-stage reduced-space optimal interpolation procedure, and the superposition of quality-improved gridded sea surface temperature observations onto reconstructions, based on historical ice concentrations at the Earth’s poles^65^. The product was developed by the Met Office Hadley Centre (https://hadobs.metoffice.com/) for use in climate monitoring and modeling, and is freely distributed at a monthly- one-degree resolution by the Environmental Research Division’s Data Access Program of the National Oceanic and Atmospheric Administration (https://coastwatch.pfeg.noaa.gov/erddap/griddap/erdHadISST.html), for which 14 SST prediction points corresponded to the Gulf of California (Fig. 1).

### Habitat-based population trend

We fitted a regression model of the 42 annual predictions of California sea lion population size as a function of SSTa at an annual basis, that is, only for the predicted values for July each year, the beginning of the breeding season when the counts were made. For this, we used the means and standard deviations of the posterior distributions of these two parameters, estimated by the two models described above. We tested for incremental polynomial degrees for the regression, but also for pairwise combinations of models through a mixing parameter^66^, which can add flexibility to the curve and increase the explained variability. The best fit was chosen based on the lowest DIC. The results of this function allowed us to calculate geometrically a range of optimum habitat, which was defined as the range of SSTa values at which the population size predictions reached the maximum at the lower and upper 95%-credible interval limits of the curve (Fig. 5). The same approximation was used to calculate the SSTa value at which the population size would reach half of its multi-decadal maximum.

### Algebraic and JAGS code details

For the numerical reconstruction of the California sea lion population size in the Gulf of California, all colony counts (*C*) available (*i*) were assumed to come from a Poisson likelihood with a logarithmic link, whose parameter (*λ*) was a function of the year (*y*) at each reproductive colony (*j*), which had fixed effects on the relation:

1 $$C_{i} = Poisson(\log [\alpha_{i,j} + \alpha 1_{i,j} \cdot y_{i} + \alpha 2_{i,j} \cdot y^{2} ])$$

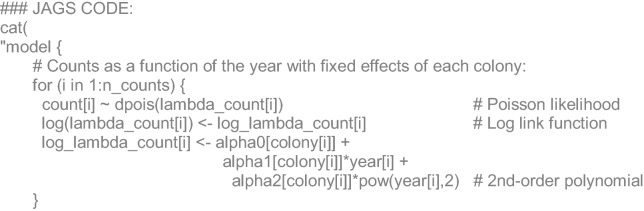
For each reproductive colony (*j*), boat-based visual counts (*V*) in 2016 came from a Binomial likelihood, with a mean proportion of detected animals (*P*_*det*_) respect to the total (*T*_*drone*_) (i.e. drone-based). The complement proportion of the former corresponds to un-detected animals (*P*_*und*_):

2 $$\begin{aligned} V_{j} & = Binomial(P\det ,Tdrone_{j} ) \\ Pund & = 1 - P\det \\ \end{aligned}$$

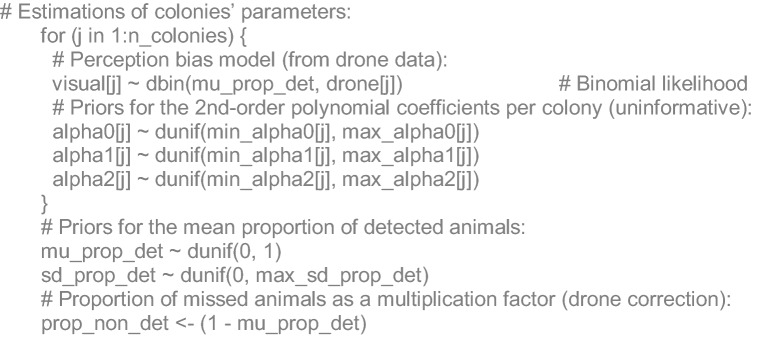
For each survey (*k*) with available information of pups, non-pup counts (*Cnp*) were stated to come from a Binomial likelihood with a mean proportion (*P*_*np*_) respect to the total (*T*):

3 $$Cnp_{k} = Binomial(Pnp,T_{k} )$$

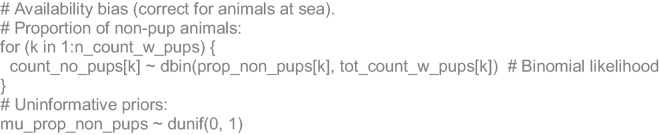
 For each reference (*l*) of the proportions published of adult animals at sea (*P*_*ats*_), a normal likelihood was stated with unknown mean (*µ*_*ats*_) and standard deviation (*σ*_*ats*_):

4 $$Pats_{l} = N(\mu_{ats} ,\sigma_{ats}^{2} )$$

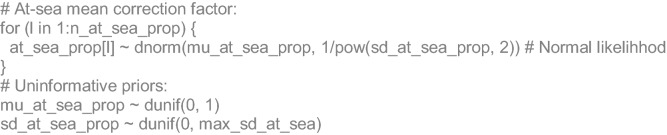
 The only total count (*TC*) available encompassing all reproductive and non-reproductive colonies was stated as the second parameter of a binomial likelihood whose number of animals at the 16 non-reproductive colonies (*N*_*nrc*_) represents a mean proportion *P*_*nrc*_:

5 $$N_{nrc} = Binomial(P_{nrc} ,TC)$$

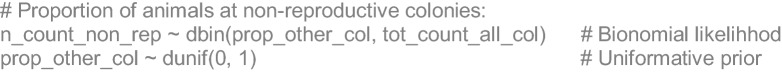
 The annual (*y*) predictions of animal abundance (*A*) for the 13 reproductive colonies (*r*) summed were derived from the function in Eq. 1, adding the proportion of undetected animals (*P*_*und*_) in visual surveys (i.e. drone-based correction) (Eq. 2):

6 $$A_{y,r} = \exp [\alpha 0_{r} + \alpha 1_{r} \cdot Y_{y} + \alpha 2_{r} \cdot Y_{y}^{2} ] \cdot (1 + P_{und} )$$

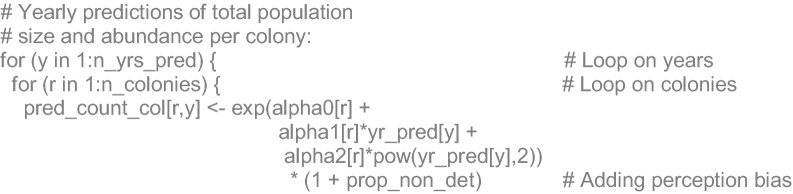
 For each year (*y*), a number of adult animals (*Ad*) was estimated at each colony (*r*), using the mean proportion of non-pup animals (*P*_*np*_) defined in Eq. 3:

7 $$Ad_{y,r} = A_{y,r} \cdot Pnp$$

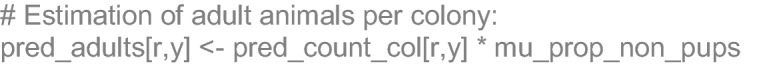


The latter estimate was used to include the correction factor for at-sea animals, not available during the counts, and to obtain the completely corrected annual estimates of abundance (*CA*) for each colony (*r*):

8 $$CA_{y,r} = A_{y,r} + Ad_{y,r} \cdot \mu_{ats}$$

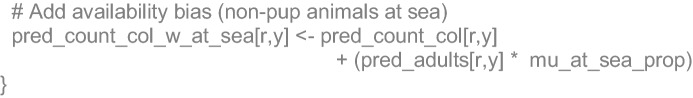
 The annual population sizes for the 42 years of the series (*y*) were estimated as the sum of those of the 13 reproductive colonies (*i*), plus the proportion of animals at non-reproductive colonies (*Pnrc*):

9 $$N_{y} = (1 + Pnrc) \cdot \sum\limits_{r = 1}^{r = n} {CA_{y,r} }$$

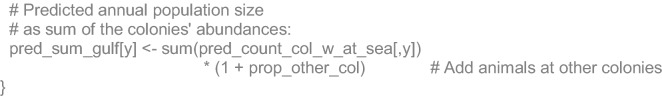


As secondary derived quantities, we estimated the percentage of decadal (*n*) changes in the population size and the main percentage decrease from the highest to the lowest:
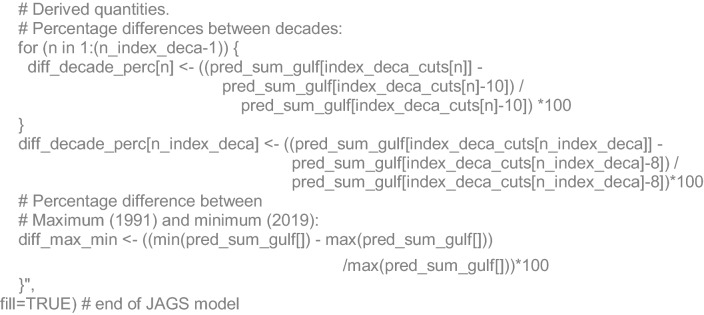
 The analysis of sea surface temperature began by stating the monthly (*m*) 25-year running means of sea surface temperature (*SST*) to come from a normal likelihood with the 100-year mean (*µ*_*SST*_) and known standard deviations (*σ*_*SST*_; calculated along with the running means). The former was subtracted from the observations to estimate the anomalies (*SSTa*):

10 $$\begin{aligned} SST_{m} & \sim N(\mu_{SST} ,\sigma_{{SST_{m} }}^{2} ) \\ SS{\text{T}} a_{m} & = SST_{m} - \mu_{SST} \\ \end{aligned}$$

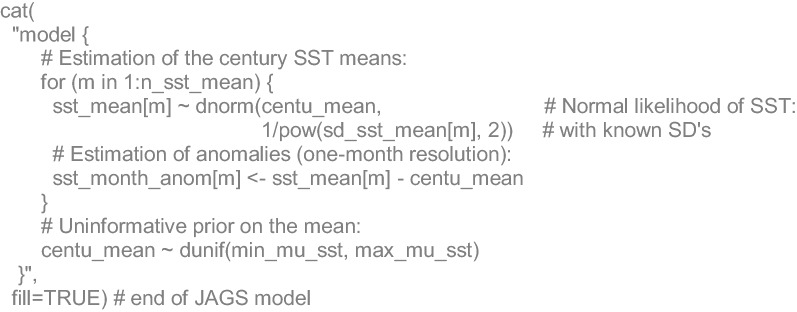
 Finally, the ecological model stated that the estimated annual (*y*) populations sizes (*N*) followed a Normal likelihood, whose means were a function of the SST anomalies (*SSTa)* (only for July estimations). The function with the lowest DIC was a mixture between a parabola and a second-order polynomial with *θ* coefficients, through a mixing parameter (*ω*). The *SSTa* estimations were also stated to come from a Normal likelihood with a mean (*µ*_*SSTa*_) and known standard deviations (*σ*_*SSTa*_):

11 $$\begin{aligned} N_{y} &\sim \,N(\mu_{{N_{y} }} ,\sigma_{y}^{2} ) \hfill \\ SSTa_{y} &\sim \,N(\mu_{SSTa} ,\sigma_{{SSTa_{y} }}^{2} ) \hfill \\ \log (\mu_{{N_{y} }} ) &= (1 - \varpi ) \cdot (\theta_{0} + \theta_{1} \cdot SSTa_{y} + \theta_{2} \cdot SSTa_{y}^{2} ) + \varpi \cdot \left( {\theta_{0} + \theta_{2} \cdot SSSSTa_{y}^{2} } \right) \hfill \\ \end{aligned}$$

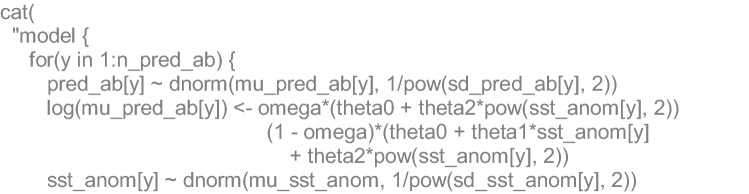
 The estimation of the variance explained by this model, the Bayesian R-squared, was based on a vector of errors (i.e. the observed minus the predicted):

12 $$R^{2} = \frac{{\sigma_{{\mu_{SSTa} }}^{2} }}{{\sigma_{{\mu_{SSTa} }}^{2} + \sigma_{{N_{SSTa} - \mu_{SSTa} }}^{2} }}$$

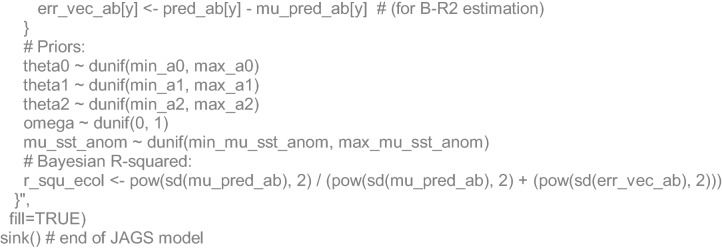


## Supplementary information

Supplementary file 1 (RData 19 kb)

Supplementary file 2 (PDF 154 kb)

## Data Availability

The authors assure the technical replication of all the analyses of our study by providing the original biological data (i.e. animal counts) in Supplementary Information Table S1, as well as a link within the Methods section granting access to the environmental data (i.e. the SST; https://coastwatch.pfeg.noaa.gov/erddap/griddap/erdHadISST.html). All the detailed code we built and the equations we used within the analyses, fully replicable in the R language, are given in the Methods section. Also, all the data necessary for running the three models is provided fully arranged as three R data list objects in Supplementary Information File S1. This saves all the programming needed to process and arrange the data.
