# Supplementary material for: The demographic decline of a sea lion population followed multi-decadal sea surface warming
Source: Sci Rep. 2020 Jun 26;10:10499. doi: 10.1038/s41598-020-67534-0 (PMC7320179; doi:10.1038/s41598-020-67534-0)
Supplement: Supplementary file 2 — (PDF 154 kb) [file 41598_2020_67534_MOESM2_ESM.pdf]

Supplementary Information Table

The demographic decline of a sea lion population followed multi-decadal sea surface warming

Karen Adame[a,b], Fernando R. Elorriaga-Verplancken[b], Emilio Beier[a], Karina Acevedo-Whitehouse[c], and Mario A. Pardo[d,1]

a. Centro de Investigación Científica y de Educación Superior de Ensenada (CICESE), Unidad La Paz, Laboratorio de Macroecologia Marina. La Paz, Baja California Sur 23050, México.
b. Instituto Politécnico Nacional, Centro Interdisciplinario de Ciencias Marinas, Avenida Instituto Politécnico Nacional s/n, Colonia Playa Palo de Santa Rita, Apartado Postal 592, CP 23096, La Paz, Baja California Sur, Mexico.
c. Unit for Basic and Applied Microbiology. School of Natural Sciences. Autonomous University of Queretaro, Queretaro 76230, Mexico.
d. Consejo Nacional de Ciencia y Tecnología (CONACYT) – CICESE, Unidad La Paz, Laboratorio de Macroecología Marina. La Paz, Baja California Sur 23050, Mexico.
1. Corresponding author. Email: mpardo@cicese.mx. Phone: +52(1)612-1213031 ext. 28116. ORCID: 0000-0003-1248-3399.

Table S1 - California sea lion counts, used in this study, in the reproductive colonies of the Gulf of California, during the breeding season. The colonies are numbered from north to south, as portrayed in Fig. 1 (see references in the Main Manuscript).

Year	Reproductive colony	Total count (visual)	Pup count (visual)	Total count (drone)	Pup count (drone)	Study
**1978**	13. Los Islotes	165	40	-	-	Aurioles & Le Boeuf (1991)
**1979**	2. San Jorge	3253	1030	-	-	Le Boeuf et al. (1983)
	2. San Jorge	3253	130	-	-	Aurioles & Le Boeuf (1991)
	4. Granito	1102	337	-	-	Le Boeuf et al. (1983)
	5. Cantiles	1810	446	-	-	Le Boeuf et al. (1983)
	6. Machos	1316	168	-	-	Le Boeuf et al. (1983)
	9. San Esteban	3961	820	-	-	Le Boeuf et al. (1983)
	10. S.P. Martir	1634	321	-	-	Le Boeuf et al. (1983)
	11. S.P. Nolasco	846	232	-	-	Le Boeuf et al. (1983)
	13. Los Islotes	138	28	-	-	Le Boeuf et al. (1983)
	13. Los Islotes	157	39	-	-	Aurioles & Le Boeuf (1991)
**1980**	13. Los Islotes	159	38	-	-	Aurioles & Le Boeuf (1991)
**1981**	1. Consag	421	10	-	-	Le Boeuf et al. (1983)

	2. San Jorge	3344	457	-	-	Le Boeuf et al. (1983)
	5. Cantiles	1380	270	-	-	Le Boeuf et al. (1983)
	11. S.P. Nolasco	1126	125	-	-	Le Boeuf et al. (1983)
	12. Farallon	323	64	-	-	Le Boeuf et al. (1983)
	13. Los Islotes	150	46	-	-	Aurioles & Le Boeuf (1991)
**1982**	12. Farallon	702	248	-	-	Aurioles & Le Boeuf (1991)
	13. Los Islotes	177	54	-	-	Aurioles & Le Boeuf (1991)
**1983**	2. San Jorge	3663	907	-	-	Aurioles & Le Boeuf (1991)
	11. S.P. Nolasco	832	150	-	-	Aurioles & Le Boeuf (1991)
	12. Farallon	551	222	-	-	Aurioles & Le Boeuf (1991)
	13. Los Islotes	143	50	-	-	Aurioles & Le Boeuf (1991)
**1984**	3. Lobos	3117	600	-	-	Aurioles-Gamboa & Zavala (1994)
	11. S.P. Nolasco	757	127	-	-	Aurioles & Le Boeuf (1991)
	13. Los Islotes	154	49	-	-	Aurioles & Le Boeuf (1991)
**1985**	2. San Jorge	3413	791	-	-	Aurioles-Gamboa & Zavala (1994)
	13. Los Islotes	176	52	-	-	Aurioles & Le Boeuf (1991)
**1990**	2. San Jorge	6159		-	-	Maravilla et al. (2006)
	4. Granito	1387		-	-	Maravilla et al. (2006)
	5. Cantiles	1239		-	-	Maravilla et al. (2006)
	6. Machos	1507	374	-	-	Aurioles-Gamboa & Zavala (1994)
	6. Machos	1072		-	-	Maravilla et al. (2006)
	8. Rasito	355		-	-	Maravilla et al. (2006)
	9. San Esteban	5236	1938	-	-	Aurioles-Gamboa & Zavala (1994)
	9. San Esteban	4234		-	-	Maravilla et al. (2006)
	10. S.P. Martir	1450		-	-	Maravilla et al. (2006)
	11. S.P. Nolasco	1039		-	-	Maravilla et al. (2006)
	13. Los Islotes	153		-	-	Maravilla et al. (2006)
**1991**	4. Granito	1662	158	-	-	Aurioles-Gamboa & Zavala (1994)
	5. Cantiles	1310	389	-	-	Aurioles-Gamboa & Zavala (1994)
	7. Partido	912	26	-	-	Aurioles-Gamboa & Zavala (1994)
	8. Rasito	430	77	-	-	Aurioles-Gamboa & Zavala (1994)
	10. S.P. Martir	1843	464	-	-	Aurioles-Gamboa & Zavala (1994)
	11. S.P. Nolasco	1193	184	-	-	Aurioles-Gamboa & Zavala (1994)
**1993**	13. Los Islotes	291	82	-	-	Aurioles-Gamboa & Zavala (1994)
**1997**	11. S.P. Nolasco	659	171	-	-	Szteren et al. (2006)

**2002**	1. Consag	600	140	-	-	Szteren et al. (2006)
**2004**	2. San Jorge	3833	968	-	-	Szteren et al. (2006)
	3. Lobos	1950	195	-	-	Szteren et al. (2006)
	4. Granito	848	246	-	-	Szteren et al. (2006)
	5. Cantiles	634	144	-	-	Szteren et al. (2006)
	6. Machos	580	102	-	-	Szteren et al. (2006)
	7. Partido	449	82	-	-	Szteren et al. (2006)
	8. Rasito	375	55	-	-	Szteren et al. (2006)
	9. San Esteban	5666	1748	-	-	Szteren et al. (2006)
	10. S.P. Martir	1171	247	-	-	Szteren et al. (2006)
	12. Farallon	280	73	-	-	Szteren et al. (2006)
	13. Los Islotes	439	143	-	-	Szteren et al. (2006)
**2012**	13. Los Islotes	428	84	-	-	Adame et al. (2017)
**2013**	13. Los Islotes	470	120	-	-	Adame et al. (2017)
	13. Los Islotes	353	78	-	-	Adame et al. (2017)
	13. Los Islotes	448	40	-	-	Adame et al. (2017)
**2014**	13. Los Islotes	515	155	-	-	Adame et al. (2017)
	13. Los Islotes	458	57	-	-	Adame et al. (2017)
**2015**	13. Los Islotes	292	9	-	-	Adame et al. (2017)
	13. Los Islotes	326	50	547	189	Adame et al. (2017)
**2016**	1. Consag	279	22	486	91	This study
	2. San Jorge	1771	357	3385	1043	This study
	3. Lobos	833	101	1901	388	This study
	4. Granito	656	76	968	140	This study
	5. Cantiles	352	52	667	95	This study
	6. Machos	533	40	857	132	This study
	7. Partido	312	32	430	49	This study
	8. Rasito	153	11	194	15	This study
	9. San Esteban	2892	467	4991	1114	This study
	10. S.P. Martir	668	86	1505	274	This study
	11. S.P. Nolasco	433	39	721	147	This study
	12. Farallon	249	73	544	174	This study
	13. Los Islotes	506	86	671	188	This study
**2017**	13. Los Islotes	433	99	-	-	This study
**2018**	13. Los Islotes	400	97	-	-	This study
**2019**	13. Los Islotes	455	110	-	-	This study
